# COI Barcoding Unveils Hidden Fish Diversity and Potential Cryptic Lineages in Inner Mongolia, China

**DOI:** 10.1002/ece3.74258

**Published:** 2026-09-28

**Authors:** Yibo Zhao, Min Wang, Zhuning Liu, Jinhui Yu, Ke Ma, Xiangchao Si, Meng Zhang, Shangjin Qiu, Yikai Li, Chongnv Wang, Yongtao Tang, Baocheng Guo, Xinxin Li, Chuanjiang Zhou

**Affiliations:** ^1^ College of Life Sciences, Henan Normal University The Observation and Research Field Station of Taihang Mountain Forest Ecosystems of Henan Province Xinxiang China; ^2^ State Key Laboratory of Animal Biodiversity Conservation and Integrated Pest Management, Institute of Zoology Chinese Academy of Sciences Beijing China; ^3^ University of Chinese Academy of Sciences Beijing China; ^4^ College of Fisheries Henan Normal University Xinxiang China

**Keywords:** cytochrome c oxidase I, freshwater fishes, ichthyofauna, molecular operational taxonomic units, species delimitation

## Abstract

The freshwater fish fauna of the Inner Mongolia Plateau remains poorly characterized at the molecular level despite its unique biogeographic setting, which combines the Yellow River (Huang He) drainage with multiple isolated endorheic basins. To address this knowledge gap, we conducted systematic sampling at 99 sites across major river systems in Inner Mongolia from 2021 to 2025 and generated 984 cytochrome c oxidase, subunit 1 gene (COI) barcode sequences, which were combined with 96 publicly available sequences to assemble a dataset of 1080 sequences. Using morphological identification together with two tree‐based species delimitation methods (mPTP and GMYC), we recovered 70 molecular operational taxonomic units (OTUs), corresponding to 62 morphologically identified species and three unidentified lineages. The detected species represent only 54.39% of historically recorded species in the region, while multiple widespread species (e.g., *Abbottina rivularis* , *Barbatula toni* , and *Rhinogobius cliffordpopei* ) exhibited substantial genetic differentiation and were partitioned into multiple independent OTUs, revealing overlooked cryptic diversity. This study demonstrates the effectiveness of COI barcoding for rapid regional fish diversity assessment and cryptic species discovery, while also highlighting the limitations of single‐locus markers in resolving recently diverged lineages. The barcode reference dataset and the cryptic lineages identified here provide critical baseline data for aquatic biodiversity conservation, fisheries management, and environmental impact assessments in this ecologically fragile region.

## Graphical abstract

Using 1080 COI barcode sequences from 99 sites across Inner Mongolia, we uncovered 70 molecular OTUs (62 morphospecies), three cryptic lineages, and deep genetic splits within widespread species. Our results provide the first regional barcode reference while revealing both the power and the limitations of single‐locus barcoding for rapid diversity assessment.

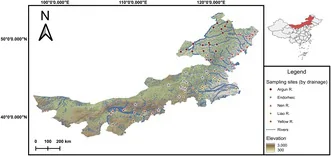

## 1 Introduction

Situated in the arid and semi‐arid region of northern China, the Inner Mongolia Plateau contains a hydrographic network with an exorheic component consisting of tributaries of the Yellow River and an endorheic component consisting of several isolated systems draining into the Hulun, Ulansuhai, Daihai, and Dalinor lakes (Zhang et al. 2008). These endorheic drainages are hydrologically isolated and often occur in high‐altitude environments characterized by extreme evaporation (Ye et al. 2024), fostering a distinctive fish fauna (Yang et al. 2023). The fish fauna in this region is relatively simple in composition but shows considerable endemism, making it a valuable system for studies of adaptive evolution and biogeography (Xu et al. 2017). However, these ecosystems are exceptionally fragile. In recent years, they have faced severe threats to aquatic biodiversity due to the dual pressures of intensified climate change and human activities—manifested primarily as river flow interruption caused by agricultural irrigation (Lian et al. 2026), heavy metal pollution of water bodies from industrial and mining activities (Li 2015), and soil erosion resulting from overgrazing and reclamation of grasslands.

Regarding fish diversity in Inner Mongolia, the most detailed and comprehensive records are from the Animal Records of Inner Mongolia (Xu 2011), which documented 114 fish species, primarily based on traditional morphological classification. As a cornerstone of baseline surveys for the region's ichthyofauna, this historical work holds significant reference value. However, traditional morphological identification has inherent limitations in distinguishing closely related species with phenotypic plasticity, identifying individuals across different life history stages, and uncovering cryptic species that are morphologically indistinguishable (Lu et al. 2026). Furthermore, over the past few decades, the hydrological patterns and environmental quality of Inner Mongolia have undergone profound changes. A systematic assessment based on contemporary samples is still lacking, making it difficult to determine whether historically recorded species persist, whether their distributions have shifted, and how much diversity may remain overlooked under morphology alone. Although cytochrome c oxidase, subunit 1 gene (COI) barcoding technology has been widely applied in fish diversity studies of large river systems such as the Yangtze River (Dong et al. 2025) and the mainstream of the Yellow River (Zhou et al. 2025), and its efficacy for rapid species identification and cryptic species discovery has been validated, its application in the unique geographical unit of Inner Mongolia—whether in the upper reaches of the Yellow River or in inland closed drainage systems—remains limited and has mostly focused on specific lake systems (Zhang et al. 2024). To our knowledge, a region‐wide molecular assessment of fish diversity across the major river systems of Inner Mongolia remains lacking.

It is noteworthy that the Yellow River section within Inner Mongolia differs markedly from the middle and lower reaches in terms of ecology. Upon entering Inner Mongolia from Ningxia, the Yellow River forms the meandering “Ji Zi Wan” plain channel, where the flow is relatively gentle (Mi et al. 2024). This section harbors endemic fish adapted to plateau or northern cold‐water environments, such as certain species of the subfamily Schizothoracinae (Li et al. 2024; Li and Guo 2020) and the genus *Triplophysa* (Yang et al. 2023). In contrast, inland lakes of Inner Mongolia, such as Hulun Lake and Daihai Lake, have experienced long‐term hydrological isolation and may contain distinct evolutionary lineages or locally differentiated populations. However, systematic COI barcoding studies targeting these critical waters remain relatively scarce. Therefore, a systematic molecular assessment is needed to update our understanding of fish diversity in this region.

To address this knowledge gap, we conducted systematic sampling across the major river systems of Inner Mongolia and performed DNA barcoding analysis based on the mitochondrial gene COI. This study aimed to: (1) establish a local COI barcode dataset for fishes in Inner Mongolia, laying the groundwork for a future regional reference database; (2) assess the current fish species diversity in the area and its discrepancy with historical records; and (3) examine the presence of cryptic or potentially new species. By integrating morphological identification with molecular species delimitation methods, this study not only provides the first large‐scale molecular assessment of fish diversity in this region but also offers critical baseline data for aquatic biodiversity conservation, fisheries management, and environmental impact assessments in this ecologically fragile area.

## 2 Materials and Methods

### 2.1 Sample Collection and Morphological Identification

To comprehensively capture the current status of fish diversity in Inner Mongolia, a total of 984 fish samples were collected from 99 sampling sites across the major surveyed river systems of Inner Mongolia between 2021 and 2025 (Figure 1 and Table S1). Fish samples were morphologically identified following the information provided in the Animal Records of Inner Mongolia (Xu 2011). Specimens that did not match the descriptions in published studies and could not be identified were classified as unidentified taxa. Tissue samples for DNA extraction were preserved in 95% ethanol, while some voucher specimens were fixed in 10% formaldehyde solution. All experimental protocols involving animals were approved by the Animal Research and Ethics Committee of Henan Normal University and conformed to the Laboratory Animal Guidelines for the Ethical Review of Animal Welfare (GB/T 35892–2018). All taxonomic identifications and higher‐level classifications were verified and updated following Eschmeyer's Catalog of Fishes (Fricke et al. 2026), the standard reference for fish taxonomy maintained by the California Academy of Sciences. Scientific names and their synonyms were cross‐checked against the Catalog to ensure nomenclatural accuracy.

**FIGURE 1:**
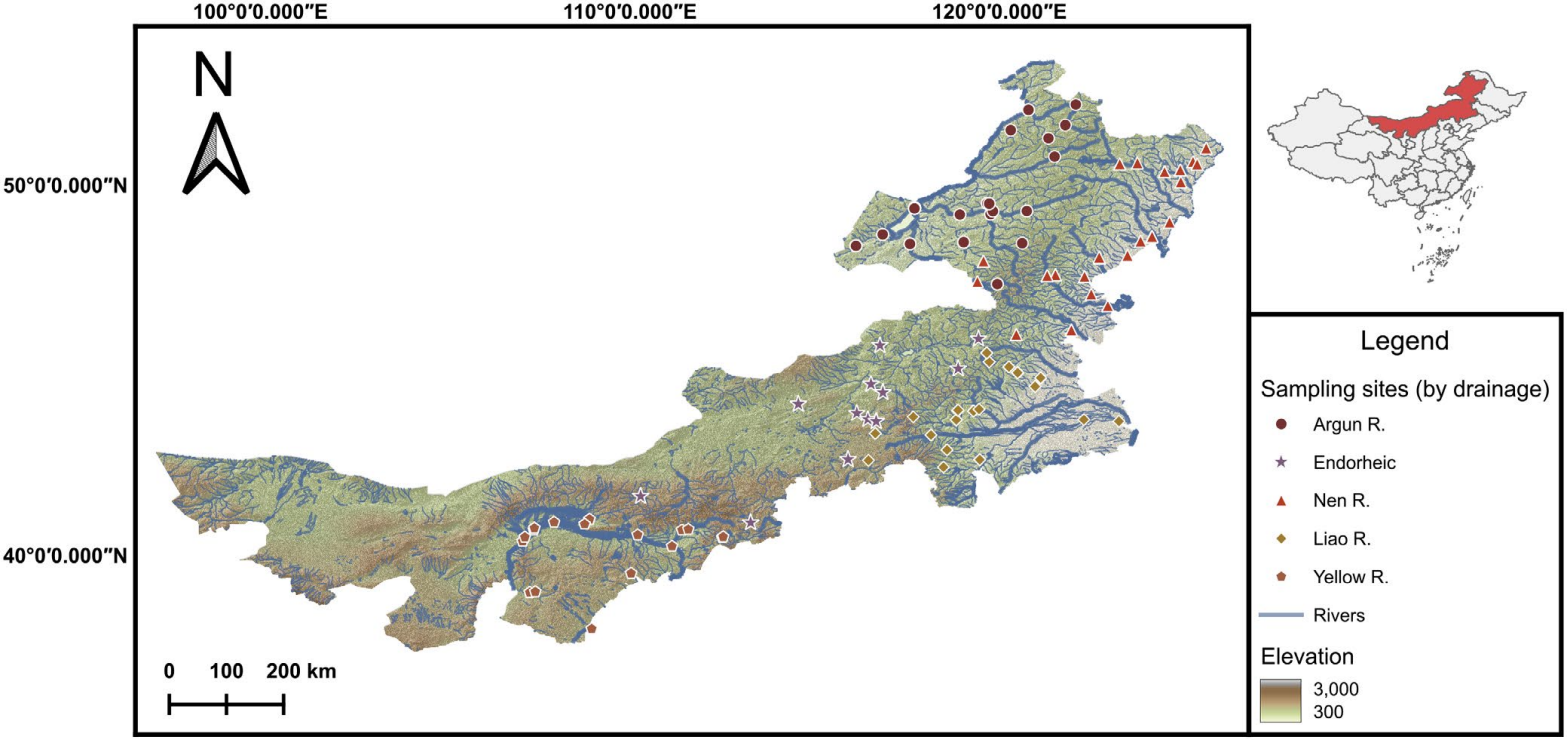
Collection sites involved in this study.

### 2.2 Obtaining DNA Sequences

All fish DNA was extracted from fin and muscle tissues using the high‐salt extraction protocol (Wang et al. 2001). The integrity of the extracted DNA was assessed by agarose gel electrophoresis using an electrophoresis apparatus (Shanghai Peiqing Technology Co. Ltd., China), and its concentration was estimated using a Nanodrop 2000 (Thermo Fisher Scientific, USA) spectrophotometer; the extracted DNA samples were stored at −20°C for subsequent use. The mitochondrial COI gene was selected as the molecular marker. The primer sequences used were as follows: COIF 5′‐TCA ACC AAC CAC AAA GAC ATT GGC AC‐3′ and COIR 5′‐TAG ACT TCT GGG TGG CCA AAG AAT CA‐3′ (Wu 2020). PCR amplification was performed in a 25 μL reaction volume containing 13 μL of 2× SanTaq PCR Master Mix (Sangon Biotech, Shanghai, China), 0.5 μL each of forward and reverse primers (100 μM/L), 1 μL of template DNA, and 10 μL of nuclease‐free water. The PCR amplifications were performed in a thermal cycler (Hangzhou Bioer Technology Co. Ltd., China) under the following conditions: initial denaturation at 94°C for 5 min; 30 cycles of denaturation at 94°C for 30 s, annealing at 54°C for 30 s, and extension at 72°C for 1 min; followed by a final extension at 72°C for 10 min, and finally held at 4°C. After PCR products were verified by agarose gel electrophoresis (exhibiting single, clear bands of appropriate intensity), qualified products were purified by Sangon Biotech (Shanghai) Co. Ltd. and sequenced bidirectionally using an Applied Biosystems 3730XL sequencer (Thermo Fisher Scientific, USA) with the BigDye Terminator v3.1 kit (Thermo Fisher Scientific, USA). Chromatograms were checked manually, and forward and reverse reads were assembled into consensus sequences. Consensus sequences were aligned and trimmed to a uniform length of 486 bp using MEGA 11.0 (Tamura et al. 2021). Using the newly obtained sequences as queries, we retrieved 96 related COI sequences from the NCBI nucleotide database via BLAST searches. For each query sequence, the top 3–4 hits with the highest similarity and unambiguous taxonomic assignment were selected, prioritizing sequences from the same or closely related genera to maximize coverage of the target lineages. This ensures transparency and reproducibility of our sequence selection process.

### 2.3 Analysis of Genetic Distance

Pairwise genetic distances were calculated using MEGA 11 (Tamura et al. 2021) based on the Kimura 2‐parameter (K2P) model (Kimura 1980). The analysis parameters were set as follows: the analysis was limited to pairwise comparisons of taxa; substitution types included transitions and transversions; rates per site were assumed to be uniform; gaps/missing data were handled using pairwise deletion; and variance was estimated via 2000 bootstrap replicates. Based on the resulting K2P distance matrix, and following morphological identification and OTU delimitation, custom scripts were written using the R software (R Core Team 2023) with the dplyr and ggplot2 packages to calculate the maximum intraspecific genetic distance (MaxIntra) and the minimum interspecific genetic distance (MinInter) first for morphologically identified species and then for the final OTU delimitation scheme. To visually assess the presence of a barcoding gap (Meyer and Paulay 2005), a scatter plot of MaxIntra versus MinInter was generated (Figure 2), with a diagonal line (*y* = *x*) added as a reference; points falling below the diagonal indicate that the maximum intraspecific distance exceeds the minimum interspecific distance, signifying overlap. In addition, density curves of intraspecific and interspecific genetic distances were plotted to illustrate their overall distribution patterns (Figure 3).

**FIGURE 2:**
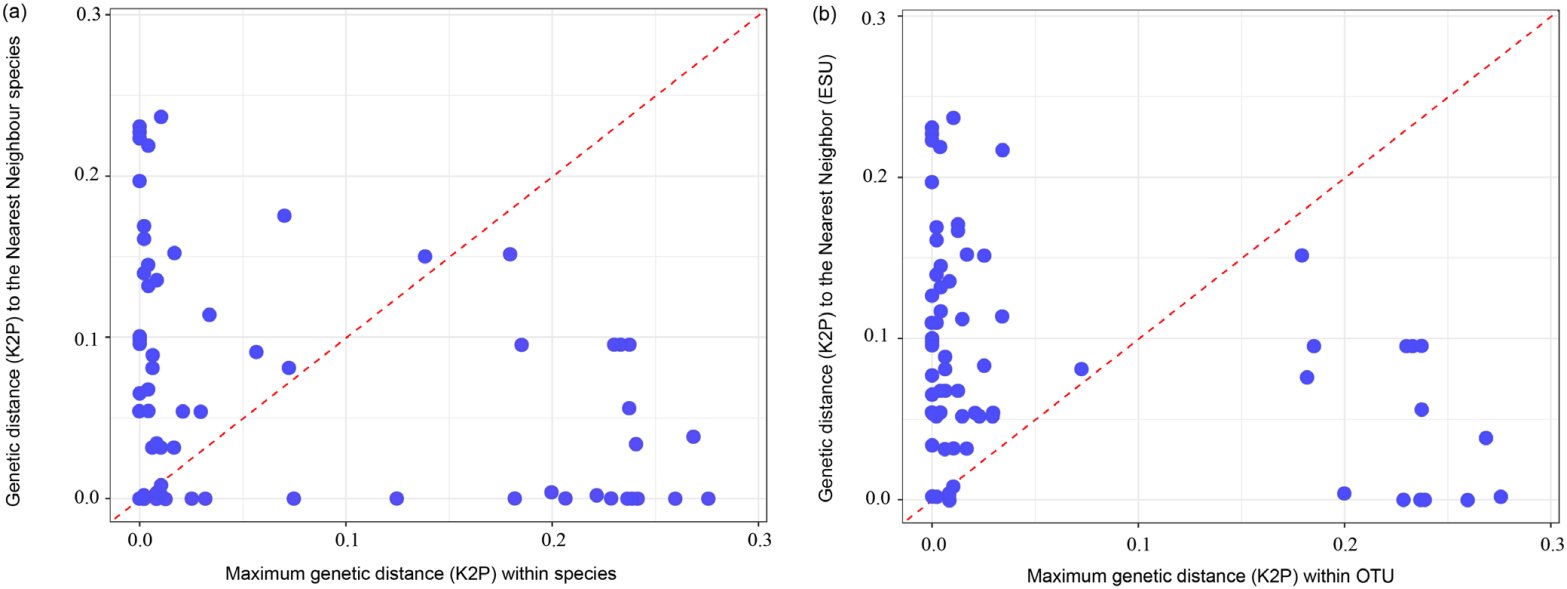
(a) Relationships between the maximum intraspecific and nearest neighbor K2P genetic distances among species. (b) Relationships between the maximum intraspecific and nearest neighbor K2P genetic distances when accounting for multiple OTUs within species.

**FIGURE 3:**
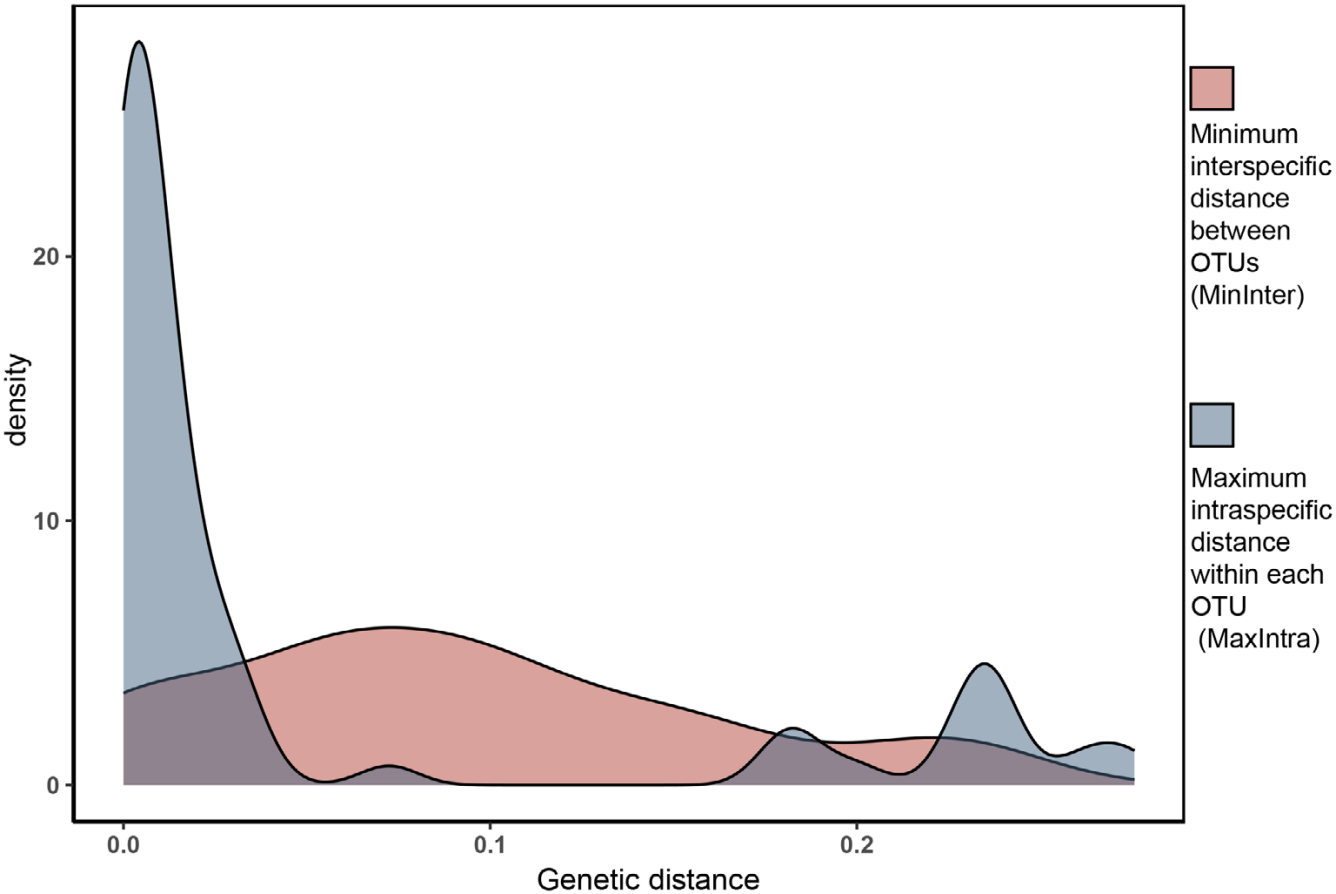
Density distribution curves of maximum intraspecific (Max Intra, blue) and minimum interspecific (Min Inter, red) K2P genetic distances based on the consensus OTU scheme. The overlap between the two curves indicates the absence of a clear barcoding gap.

### 2.4 Species Delimitation

To distinguish between species delimitation results derived from different methods (sequence‐based delimitation versus traditional morphological examination), we followed the recommendation of Hubert and Hanner (2015) to clearly differentiate between the corresponding concepts within the same analytical framework. Accordingly, we defined species units identified through DNA sequence data as operational taxonomic units (OTUs)—that is, diagnosable molecular lineages (Avise 1994; Moritz 1994; Vogler and DeSalle 1994). We employed two species delimitation methods: the multi‐rate Poisson tree process (mPTP) (Kapli et al. 2017) and the general mixed Yule‐coalescent (GMYC) model (Fujisawa and Barraclough 2013). A final delimitation scheme was established based on a 50% consensus among the methods. A maximum likelihood (ML) tree was constructed using the RAxML (Stamatakis 2006) with default parameters under the GTR + F + I + G4 model, which served as the basis for subsequent mPTP analysis. The fully resolved, ultrametric gene tree required for GMYC analysis was generated using the BEAST software package (Bouckaert et al. 2019). First, ALTER (Glez‐Pena et al. 2010) was used to collapse duplicate sequences into haplotypes. A Markov chain of 150 million iterations was run using a strict‐clock model with a divergence rate of 1% per million years (Bermingham et al. 1997) for the purpose of generating an ultrametric tree for GMYC analysis. To ensure stability of the analytical results, the first 15 million generations were discarded as burn‐in, and trees were sampled from the remaining iterations, retaining one‐third of the sampled phylogenetic trees. Finally, we used TreeAnnotator v2.7.3 (Bouckaert et al. 2019) to integrate the retained trees and establish the maximum clade credibility tree. Convergence of the phylogenetic tree was assessed using the Tracer v1.7.2, with all effective sample size (ESS) values exceeding 200, indicating satisfactory convergence. Subsequently, based on the maximum clade credibility ultrametric tree generated, GMYC analysis was performed in R using the splits package. To facilitate visualization of haplotype distribution, we also constructed a separate ML tree using the collapsed haplotypes (Figure S1), although all species delimitation analyses were performed on the full dataset.

## 3 Results

We established a COI barcode database comprising 1080 sequences, including 984 newly obtained sequences from 99 sampling sites and 96 sequences downloaded from NCBI (Table S1). This database encompasses 10 orders, 19 families, 45 genera, and 62 species. Classification following Eschmeyer's Catalog of Fishes (Fricke et al. 2026). Among these, Cypriniformes accounted for 45 species, Gobiiformes for four species, and Anabantiformes, Beloniformes, Perciformes, Salmoniformes, and Siluriformes were each represented by two species, while Cyprinodontiformes, Gadiformes, and Osmeriformes each comprised a single species. Additionally, we recovered three unidentified lineages, of which one may represent an undescribed species. The number of sequences per species ranged from 1 to 112, distributed across up to 18 county‐level cities within five prefecture‐level cities (Table S1). After collapsing identical sequences into haplotypes using the ALTER, a total of 254 distinct haplotypes were identified across all samples.

We corrected the taxonomic information for freshwater fish reported in the Animal Records of Inner Mongolia using the Eschmeyer's catalog (Fricke et al. 2026), yielding a total of 114 recorded freshwater fish species, for which a complete list with taxonomic hierarchy and reported distributional ranges is provided in Table S4. The number of freshwater fish species identified in the present study accounts for 54.39% of the total species documented in the historical literature.

The genetic distance analysis revealed that the maximum intraspecific distance was 0.276 ( *Carassius auratus* ), while the maximum nearest‐neighbor distance was 0.237 ( *Macropodus ocellatus* ). The average value of the maximum intraspecific distance was 0.077, and the average value of the nearest‐neighbor distance was 0.075. On average, the nearest‐neighbor distance was 9.36‐fold greater than the maximum intraspecific distance, with the ratio ranging from 0 to 82.0. Genetic distance analysis revealed that 28 morphologically identified species showed a minimum interspecific distance of less than 1%, of which 15 species exhibited zero distance to their nearest neighbors (Table S2). Conversely, 28 species showed a maximum intraspecific distance exceeding 2%, with some reaching as high as 27.5% (e.g., *Carassius auratus* ). Notably, these two sets largely overlap: species with exceptionally high intraspecific divergence often also show low or zero interspecific distances, reflecting the breakdown of a clear barcoding gap and suggesting the presence of cryptic diversity within morphologically defined taxa. For 27 species, the maximum intraspecific genetic distance was greater than their minimum interspecific genetic distance. Fifteen species displayed a minimum interspecific genetic distance of zero (e.g., *Hemiculter leucisculus* and *Saurogobio dabryi* ), indicating that these species share identical haplotypes in the COI fragment with at least one other species (Table S2). The density distribution curves of intraspecific and interspecific genetic distances (Figure 3) revealed pronounced overlap, indicating the absence of a clear barcoding gap even under our consensus OTU scheme. Specifically, for a substantial proportion of OTUs, the maximum within‐OTU divergence (Max Intra) equaled or exceeded the minimum distance to the nearest neighboring OTU (Min Inter). This pattern reflects the inherent limitation of single‐locus COI barcoding in resolving recently diverged or hybridizing lineages—a phenomenon widely recognized in cypriniform fishes (Chen et al. 2017)—and underscores the necessity of integrating multiple tree‐based delimitation methods (mPTP and GMYC) rather than relying on a single genetic distance threshold.

The two sequence‐based species delimitation methods yielded different numbers of OTUs, both of which exceeded the number of morphologically identified species. The results showed that mPTP generated 68 OTUs, while GMYC generated 80 OTUs. A total of 62 morphological species were identified in this study, and the consensus delimitation scheme produced 70 molecular OTUs, including three OTUs corresponding to unidentified lineages. The Bayesian phylogenetic tree (Figure 4) comprised 1080 COI sequences, including 984 newly generated in this study and 96 retrieved from GenBank, representing 62 morphologically identified species and three unidentified lineages (Table S1). The species delimitation results from mPTP and GMYC, along with the consensus scheme, were mapped onto the tree (Figure 4): cyan bars indicate morphological species assignments; light orange (mPTP), dark orange (GMYC), and red (consensus) bars show the molecular OTU schemes.

**FIGURE 4:**
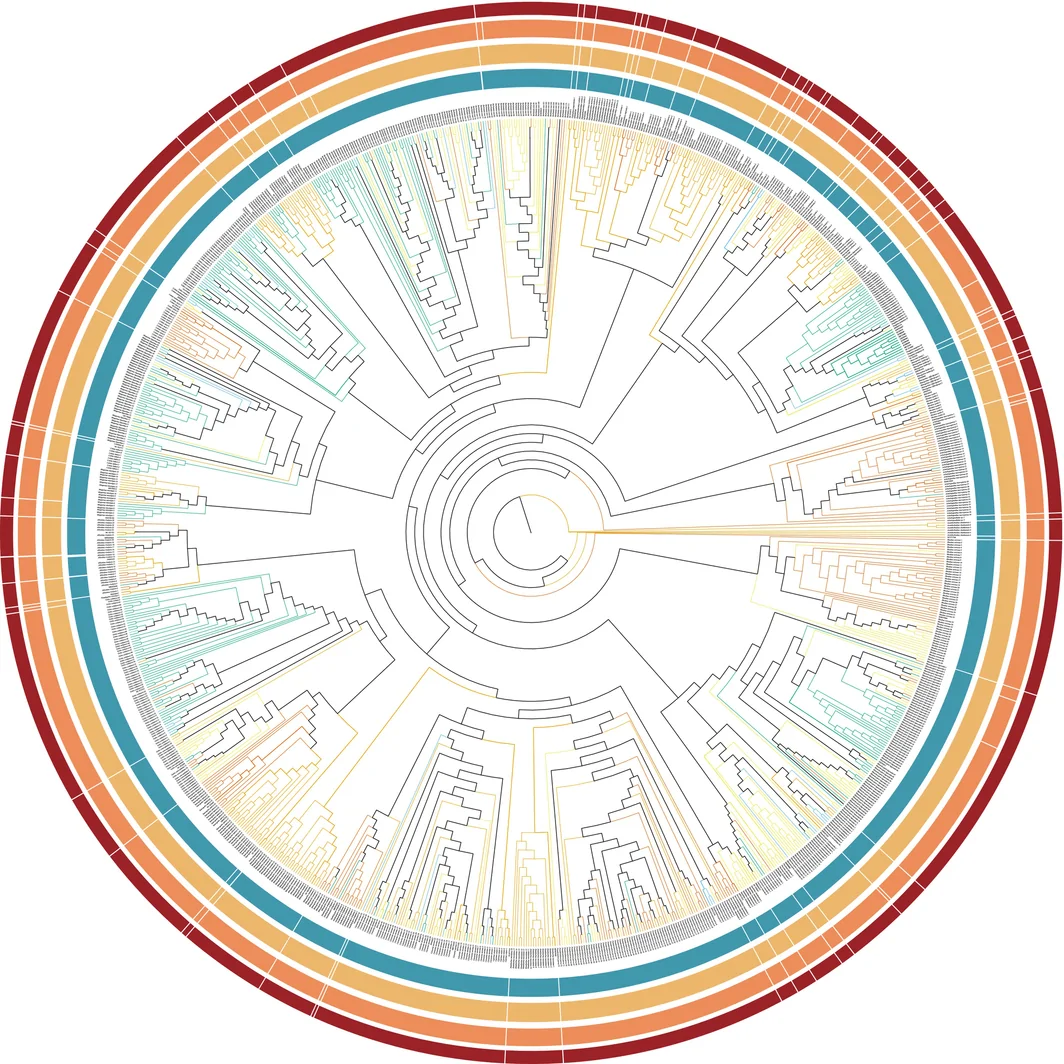
Bayesian phylogenetic tree of the 1080 COI barcodes. Terminal branches are color‐coded according to the drainage system of origin: Yellow River (orange), endorheic basins (sky blue), Liao River (teal), Nen River (yellow), and Argun River (red). Species morphological assignments are indicated by cyan bars, with mPTP (light orange), GMYC (dark orange), and consensus (red) OTU delimitation schemes shown alongside. An index cross‐referencing species names with their corresponding codes in the tree is provided in Table S5.

The mPTP method delineated 68 OTUs (numbered 1–68 in Table S1), while GMYC yielded 80 OTUs (numbered 1–80). The 50% consensus scheme recovered 70 OTUs (numbered 1–70). In most cases, the three delimitation methods were congruent for well‐differentiated species. However, discordance was observed in several taxa: for example, *Rhynchocypris lagowskii* was assigned to a single consensus OTU (OTU3) but was split into two GMYC OTUs (OTU3 and OTU4); *Cobitis melanoleuca* was assigned to a single consensus OTU (OTU31) but was split into two GMYC OTUs (OTU34 and OTU35); and *Pseudogobio esocinus* was split into two GMYC OTUs (OTU23 and OTU24) despite forming a single mPTP and consensus OTU (OTU21).

Overall, 56 of the 62 morphologically identified species exhibited a one‐to‐one correspondence with the consensus OTUs (80% congruence rate). The remaining four morphological species were subdivided into multiple consensus OTUs: *Abbottina rivularis* was split into three OTUs (OTU22, OTU23, and OTU24), *Barbatula toni* into three OTUs (OTU36, OTU37, and OTU38), *Rhinogobius cliffordpopei* into two OTUs (OTU66 and OTU67), and *Gnathopogon strigatus* into two OTUs (OTU15 and OTU16). These splitting events are visualized as multiple red bars under a single cyan bar. In addition, the three unidentified lineages formed independent consensus OTUs (OTU14, OTU28, and OTU70), which were placed as terminal branches in the tree, further supporting their recognition as distinct molecular lineages. The congruence rate between morphological species and OTUs was 80%, with 56 morphological species exhibiting a one‐to‐one correspondence with OTUs. The remaining four morphological species were further subdivided into multiple OTUs (i.e., “morphospecies splitting”), with a maximum of three OTUs per species. Three unidentified samples formed independent OTUs (OTU14, OTU28, and OTU70). Among these, OTU14 was collected from the Ulansuhai Lake area of the Yellow River system and showed the highest sequence similarity (93%) to the genus *Gnathopogon*; OTU28 and OTU70 were collected from the Dahei River basin (a tributary of the Yellow River) and the Kerulen River basin, respectively, and were most closely related to sequences from the families Cobitidae and Acheilognathidae (Table S1). Based on the final OTU delimitation scheme, the separation between the maximum intraspecific and minimum interspecific genetic distance improved, although a clear barcoding gap was still absent (Figure 4).

In this study, *Abbottina rivularis* , *Barbatula toni* , and *Rhinogobius cliffordpopei* occupied multiple OTUs (Table S1). We also identified three unidentified lineages and distinguished some of them from existing species.

## 4 Discussion

The completeness of a regional biodiversity survey is inherently limited by its spatial coverage. Based on molecular operational taxonomic unit (OTU) delimitation, this study identified 70 OTUs, corresponding to 62 morphologically identified species—a number that accounts for only 54.39% of the historical records documented in the Animal Records of Inner Mongolia (Xu 2011). However, this comparison should be interpreted cautiously, because the historical and contemporary datasets differ in spatial coverage, time period, and identification approach. Several factors may account for this discrepancy. For instance, habitat alterations may have driven population declines or range contractions in certain species, rendering them less detectable in our surveys. This substantial discrepancy may point to two non‐mutually exclusive explanations: first, some species from historical records may have experienced local extinction due to hydrological alterations and habitat degradation over recent decades, or they may be distributed exclusively in areas not covered by this survey; second, and more crucially, traditional morphological methods inherently possess.

Limitations in distinguishing closely related species and identifying cryptic species. Recent applications of environmental DNA (eDNA) technology in typical lakes of Inner Mongolia have demonstrated higher detection efficiency than traditional surveys—species detection at single sites reached up to 1.25 times that of traditional methods (Zhang et al. 2024)—suggesting that historical surveys relying solely on morphological approaches may have systematically underestimated diversity. Future research urgently needs to fill gaps in unsampled regions and conduct complementary surveys in key water bodies, such as Ulansuhai Lake and Juyan Lake, using the eDNA technology to obtain more comprehensive diversity data. The final number of OTUs exceeded the number of morphologically identified species, and some unknown species were assigned to independent OTUs (Durand et al. 2017; Hajibabaei et al. 2006; Winterbottom et al. 2014; Wu et al. 2023).

The discovery of potential cryptic diversity represents one of the key findings of this study. By integrating morphology with two tree‐based species delimitation methods (mPTP and GMYC), we not only obtained OTU numbers distinct from those of morphological identification (68 and 80 vs. 62 species) but also directly identified three independent unknown species OTUs. This indicates that, even after systematic morphological surveys, the cryptic diversity of fishes in Inner Mongolia may still be severely underestimated. This finding is highly consistent with a study conducted in Mongolia (Gransee et al. 2019), which, through integrative taxonomic analysis of Nemacheilidae samples across the Mongolian Plateau, revealed multiple cryptic lineages with interspecific genetic distances ranging from 9.3% to 17.7%, far exceeding typical intraspecific variation levels. The prolonged geographical isolation of closed lakes on the Inner Mongolia Plateau may have promoted the accumulation of distinct genetic lineages. These findings highlight the profound role of the region's distinctive hydrographic architecture in shaping population differentiation in fishes.

The elevated intraspecific genetic distances observed in multiple widespread species—including *Carassius auratus* (27.5%), *Hemibarbus maculatus* (26.8%), *Saurogobio dabryi* (25.9%), *Channa argus* (24.1%), and *Misgurnus mizolepis* (22.8%)—merit further discussion. These values, while exceptional in the context of standard DNA barcoding surveys, are consistent with findings from phylogeographic studies on the same or related taxa. For example, the *Carassius auratus* complex has been shown to comprise multiple deeply divergent mtDNA lineages with late Pliocene to Pleistocene origins (Gao et al. 2012), with most genetic variation distributed among lineages rather than within populations (Cheng et al. 2020). Similarly, *Abbottina rivularis* was found to contain four major mitochondrial lineages with uncorrected pairwise distances of 9.4%–15.2% (Jang‐Liaw et al. 2019). The genus *Misgurnus* exhibits interspecific genetic distances of 10.7%–18.4%, and the genus itself is non‐monophyletic (Zhang et al. 2021). These patterns are not anomalies but rather reflect the profound role of the Inner Mongolia Plateau's distinctive hydrographic architecture—comprising both the exorheic Yellow River system and multiple isolated endorheic basins—in driving population differentiation in freshwater fishes.

Notably, widespread species such as *Abbottina rivularis* , *Barbatula toni* , and *Rhinogobius cliffordpopei* occupied multiple OTUs. Details of the OTU partitioning and geographical distribution for these splitting species are shown in Table S3. These species are broadly distributed across Inner Mongolia, and habitat fragmentation—particularly the long‐term isolation between endorheic and exorheic drainages, as well as among different tributaries of the Yellow River's “Ji Zi Wan” reach—may have facilitated genetic differentiation among populations, thereby promoting lineage structuring within nominal species.

Further analysis of the genetic distance distribution revealed a pronounced overlap in the density curves of intraspecific and interspecific distances (Figure 3). For 27 species (approximately 43.5% of the morphologically identified species), the maximum intraspecific distance exceeded the minimum interspecific distance—indicating the absence of a barcoding gap in certain lineages. In this study, 28 fish species exhibited minimum interspecific genetic distances below 1% (Table S2), with 15 of these showing zero distance, indicating haplotype sharing in the COI fragment among these species (e.g., *Hemiculter leucisculus* , *Saurogobio dabryi* ). This phenomenon is common among closely related species, recently diverged lineages (Meyer and Paulay 2005), or groups with historical hybridization (Noutsos et al. 2014), providing valuable clues for subsequent multi‐evidence integrative species delimitation. The hydrographic network of the Inner Mongolia Plateau formed relatively recently; some fish lineages may have diverged due to geographical isolation since the Late Pleistocene to Holocene, with mitochondrial genes not yet accumulating sufficient differences to form clear interspecific boundaries (Noutsos et al. 2014). Twenty‐eight fish species showed maximum intraspecific genetic distances exceeding 2%, significantly higher than conventional species thresholds (Hebert et al. 2003). This suggests that these morphospecies actually represent complexes of multiple cryptic lineages, reflecting the driving role of long‐term geographical isolation among different river systems on the Inner Mongolia Plateau in promoting population divergence. In cyprinid fishes, particularly within subfamilies such as Gobioninae, historical hybridization events may have led to the horizontal transfer of mitochondrial genes between species (Perea et al. 2016). The haplotype sharing observed between *Hemiculter leucisculus* and its closely related species in this study may represent such a case. The comparison of species delimitation methods highlights both the utility and the limitations of single‐locus barcoding in this regional fish fauna.

In this study, the numbers of OTUs generated by the two methods, mPTP and GMYC, differed (68 vs. 80), reflecting the varying sensitivity of different algorithms to phylogenetic tree topology and branch lengths. In our dataset, GMYC tends to over‐split, whereas mPTP may be more robust to heterogeneous evolutionary rates among lineages. Accordingly, this study adopted a 50% consensus scheme to enhance the reliability of the delimitation results. Genetic distance analysis further revealed an overlap in the distributions of intraspecific and interspecific distances, with no clear barcoding gap; this phenomenon was not completely eliminated even after recalculations based on OTUs. This finding underscores the inherent limitations of single‐locus COI barcoding, particularly in regional studies that encompass recently diverged evolutionary lineages. A review by Chen et al. (2017) indicated that the overall identification success rate of COI barcoding for global freshwater fishes is approximately 93%, but this proportion declines significantly in specific groups (e.g., certain families and genera within Cypriniformes) (Ward et al. 2009; Ward 2012). Therefore, for complex taxa such as *Triplophysa* and the subfamily Gobioninae, future research needs to integrate nuclear gene markers or mitochondrial genome data to elucidate their evolutionary relationships.

The findings of this study have multiple implications for fish conservation in the ecologically fragile Inner Mongolia region. First, the three genetically distinct unidentified lineages and the cryptic lineages within widespread species such as *Abbottina rivularis* represent unique evolutionary heritage and should be prioritized as Evolutionarily Significant Units (ESUs) for further conservation (Kaus et al. 2019). Second, many rare species and cryptic lineages may be highly dependent on specific habitats—such as the slow‐flowing sections of the Yellow River's “Ji Zi Wan” reach and the unique water quality of closed lakes. River flow intermittency driven by climate change (Lian et al. 2026) and the persistent impacts of human activities, including agricultural irrigation and industrial and mining pollution (Li 2015), may expose these undescribed genetic lineages to heightened extinction risks before their taxonomic status is fully resolved. Finally, given the discrepancy between historical records and current survey (only 54.39% congruence in morphospecies), the conservation status of fishes in Inner Mongolia would benefit from reassessment based on updated integrative morphological and molecular data, together with the establishment of a dynamic monitoring framework. Future research should focus on sampling gaps in the region and on integrating multiple‐gene markers and genomic approaches to resolve complex lineages, thereby providing a more comprehensive scientific basis for aquatic biodiversity conservation in this region.

This survey did not cover the Alxa League, a region belonging to the independent Hexi Corridor‐Alxa endorheic drainage. Historically, this area harbored endemic fish species such as *Hedinichthys macropterus* and *Triplophysa jiuzhaiensis* (Xu 2011; Zhao 1991). However, due to reduced inflow in the Heihe River, West Juyan Lake dried up in 1961, and East Juyan Lake desiccated in 1992 (Liao et al. 2015), leading to the local extirpation of native fish species. Following the implementation of unified water resource regulation after 2000, East Juyan Lake was re‐watered in 2003 (Jiang and Dong 2020), and fish populations have gradually recovered, although the system remains in an ecological restoration phase (Guo 2020). The absence of sampling in this area inevitably limits the representativeness of our findings for the entirety of Inner Mongolia and highlights the need for future, more comprehensive surveys that include this unique ecoregion.

## 5 Conclusions

In this study, we conducted systematic sampling of the major river systems within Inner Mongolia and obtained COI barcoding from 984 fish samples. Combined with sequences from public databases, we compiled an analytical dataset comprising 1080 sequences. Based on combined morphological identification and two molecular species delimitation methods (mPTP and GMYC), we delineated 70 molecular operational taxonomic units (OTUs), corresponding to 62 morphologically identified species, and recovered three genetically distinct unidentified lineages. The species recovered in this study accounts for approximately 54.39% of the historical records documented in the *Animal Records of Inner Mongolia*, although this comparison should be interpreted cautiously. These results highlight the need to expand sampling coverage in future studies, particularly in unsurveyed areas, and to incorporate complementary approaches such as environmental DNA to improve species detection. At the same time, the cryptic diversity uncovered in this study—including intraspecific differentiation within several widespread species and three putative new species—suggests that traditional taxonomy may have underestimated the actual fish diversity in Inner Mongolia, and the integration of molecular approaches provides a new perspective for understanding the evolutionary history of fishes in this region. Genetic distance analyses revealed significant genetic differentiation within certain widespread species (e.g., *Abbottina rivularis* , *Barbatula toni* ), forming multiple independent OTUs, which reflects the shaping effect of the Inner Mongolia Plateau's unique hydrographic architecture on fish population evolution. This study verifies the efficacy of COI barcoding for rapid assessment of fish diversity and identification of cryptic species, while also highlighting the limitations of single‐locus markers in delimiting recently diverged lineages. Future studies should integrate multi‐locus or genomic data to further resolve evolutionary relationships within complex taxa. Overall, the COI barcode dataset and cryptic lineages identified here provide critical baseline data and a scientific foundation for aquatic biodiversity conservation, fisheries management, and environmental impact assessments in Inner Mongolia.

## Author Contributions

**Yibo Zhao:** data curation (equal), formal analysis (equal), investigation (equal), methodology (equal), project administration (equal), software (equal), validation (equal), visualization (equal), writing – original draft (equal), writing – review and editing (equal). **Min Wang:** investigation (equal). **Zhuning Liu:** investigation (equal). **Jinhui Yu:** data curation (equal). **Ke Ma:** data curation (equal). **Xiangchao Si:** data curation (equal). **Meng Zhang:** data curation (equal). **Shangjin Qiu:** data curation (equal). **Yikai Li:** investigation (equal). **Chongnv Wang:** investigation (equal). **Yongtao Tang:** investigation (equal), resources (equal), writing – review and editing (equal). **Baocheng Guo:** investigation (equal). **Xinxin Li:** investigation (equal), writing – review and editing (equal). **Chuanjiang Zhou:** conceptualization (equal), funding acquisition (equal), investigation (equal).

## Funding

This work was supported by the following funding bodies: The National Science & Technology Fundamental Resources Investigation Program of China (2023FY100303, 2025FY100800).

## Ethics Statement

Authors declare that all experimental protocols involving animals were approved by the Animal Research and Ethics Committee of Henan Normal University and the Laboratory Animal Guidelines for the Ethical Review of Animal Welfare (HNSD‐2026BS‐0401) and were conducted in accordance with the research standards.

## Conflicts of Interest

The authors declare no conflicts of interest.

## Supporting information


**Figure S1:** Maximum likelihood tree of the 254 unique COI haplotypes recovered from the 1080 sequences analyzed in this study.

**Table S1:** List of the Inner Mongolia Fish in Henan Normal University.

**Table S2:** Results of genetic distance analysis.

**Table S3:** Molecular delimitation and geographical distribution of splitting species in morphospecies.

**Table S4:** Complete list of freshwater fish species historically recorded in Inner Mongolia (based on Xu, 2011), with taxonomic hierarchy and reported distributional ranges.

**Table S5:** Index of species names and corresponding codes in the phylogenetic tree (Figure 4).

## Data Availability

All collecting and sequence data are available on the Barcode of Life Data system (BOLD) under the project “IMNFISH”. The dataset has been assigned the DOI: https://doi.org/10.5883/DS‐IMNFISH.
