# Correction to “COI Barcoding Unveils Hidden Fish Diversity and Potential Cryptic Lineages in Inner Mongolia, China”

**DOI:** 10.1002/ece3.74576

**Published:** 2026-10-20

Zhao, Y. , M. Wang , Z. Liu , et al. 2026. “COI Barcoding Unveils Hidden Fish Diversity and Potential Cryptic Lineages in Inner Mongolia, China.” Ecology and Evolution 16, no. 10: e74258. 10.1002/ece3.74258.PMC1361935542812194

In the published article, the equal co‐first authorship designation for Yibo Zhao and Min Wang was omitted from the author information. A note has been added to the online version: Yibo Zhao and Min Wang contributed equally to this work.

We apologize for this error.

